# Dataset with Tactile and Kinesthetic Information from a Human Forearm and Its Application to Deep Learning

**DOI:** 10.3390/s22228752

**Published:** 2022-11-12

**Authors:** Francisco Pastor, Da-hui Lin-Yang, Jesús M. Gómez-de-Gabriel, Alfonso J. García-Cerezo

**Affiliations:** Robotics and Mechatronics Group, Escuela de Ingenierías Industriales, University of Malaga, 29071 Málaga, Spain

**Keywords:** physical human–robot interaction, grippers for physical human-robot interaction, conv-LSTM, haptic perception

## Abstract

There are physical Human–Robot Interaction (pHRI) applications where the robot has to grab the human body, such as rescue or assistive robotics. Being able to precisely estimate the grasping location when grabbing a human limb is crucial to perform a safe manipulation of the human. Computer vision methods provide pre-grasp information with strong constraints imposed by the field environments. Force-based compliant control, after grasping, limits the amount of applied strength. On the other hand, valuable tactile and proprioceptive information can be obtained from the pHRI gripper, which can be used to better know the features of the human and the contact state between the human and the robot. This paper presents a novel dataset of tactile and kinesthetic data obtained from a robot gripper that grabs a human forearm. The dataset is collected with a three-fingered gripper with two underactuated fingers and a fixed finger with a high-resolution tactile sensor. A palpation procedure is performed to record the shape of the forearm and to recognize the bones and muscles in different sections. Moreover, an application for the use of the database is included. In particular, a fusion approach is used to estimate the actual grasped forearm section using both kinesthetic and tactile information on a regression deep-learning neural network. First, tactile and kinesthetic data are trained separately with Long Short-Term Memory (LSTM) neural networks, considering the data are sequential. Then, the outputs are fed to a Fusion neural network to enhance the estimation. The experiments conducted show good results in training both sources separately, with superior performance when the fusion approach is considered.

## 1. Introduction

In recent years, Human–Robot Interaction (HRI) has become a relevant topic in robotic research. HRI studies focus on analyzing the collaboration and communication between humans and robots. Some fields in robotics have used HRI solutions in complex tasks that robots or humans can not solve individually [1]. Many solutions focus on improving task performance without reducing safety [2,3,4,5,6,7]. In surgical robotics, doctors are assisted by robots, improving their skills and reducing the risks associated with an intervention [2,3]. Collaborative robot manipulators (co-bots) are frequently used to improve productivity inside industries [4,5]. Further, some HRI studies have played a significant role in Search And Rescue (SAR) operations, such as developing systems that place sensors and monitor vital signs [6] or optimizing signal policies in rescue operations using game theoric approaches [7].

Physical Human–Robot Interaction (pHRI) is required for many applications, such as exoskeletons [8], rehabilitation [9,10], or prostheses [11]. Usually, these applications need solutions that satisfy multiple objectives, as in [12], where Genetic Algorithms (GA) are implemented. One of the ultimate goals for several scenarios involving pHRI is to achieve natural contact through touch between the participants [13]. A common definition of touch sensing is the perception of tactile and kinesthetic signals through the skin [14]. Kinesthetic is defined as two sources of information: the relative positions of the limbs and the dynamical forces produced by the muscles [15]. Haptic perception is the term used to describe the combination of tactile and kinesthetic information. Without the sensations experienced in this sense, we would be unable to perform numerous essential tasks. This necessity is also present in the field of robotics [16]. Tactile sensors have played a crucial role in this sense, enhancing the capabilities whenever integrated into robotic systems [17,18]. The most common haptic perception approaches use static tactile information only. Traditional computer vision and machine learning techniques have been used to challenge the problem of tactile object recognition [19,20]. Considering tactile data as dynamic information is an approach some researchers have considered [21,22]. Other researchers proved that haptic data could be treated as sequential data; therefore, Long-Short Term Memory (LSTM) provided excellent results in detecting the slip direction [23]. Kinesthetic data have also been proven to be useful. In [24], the interaction forces between a gripper and a human are estimated using proprioceptive information only. Moreover, an estimation of the roll angle of a wrist using kinesthetic data is presented in [25]. Despite the benefits of combining numerous haptic-based sources, only a few studies have followed this strategy. In [26], a single and unplanned grasp is performed on multiple objects, and an approach to classify them using the proprioceptive and tactile data of the gripper is presented. Further, in our previous work, we developed a fusion of haptic data to classify objects and enhance the results of the tactile and kinesthetic approaches performed separately [27].

Reacting to haptic inputs is a key component in pHRI, which typically requires a robot with tactile sensors and/or kinesthetic perception capabilities. The recognition system’s algorithm usually needs a dataset for training purposes. In recent years, several haptic datasets have been presented. In [28], Wang et al. present "TacAct", which contains tactile data from multiple subjects and differentiates types of touch actions using a convolutional neural network. On the other hand, in [29], tactile data are recorded from grasping objects with a sensed globe. A neural network is also trained to classify the objects in the dataset. Albini et al. presented a method to discriminate between touch from human and non-human hands, trained with the collected dataset [30]. Not many datasets containing both tactile and kinesthetic information are found in the literature. In [31], both sources are recorded from the NICO humanoid robot, classifying in-hand objects using various neural network approaches. Nevertheless, no regression approaches were found in the literature using tactile and kinesthetic data as the input, primarily due to the lack of haptic datasets.

**Figure 1:**
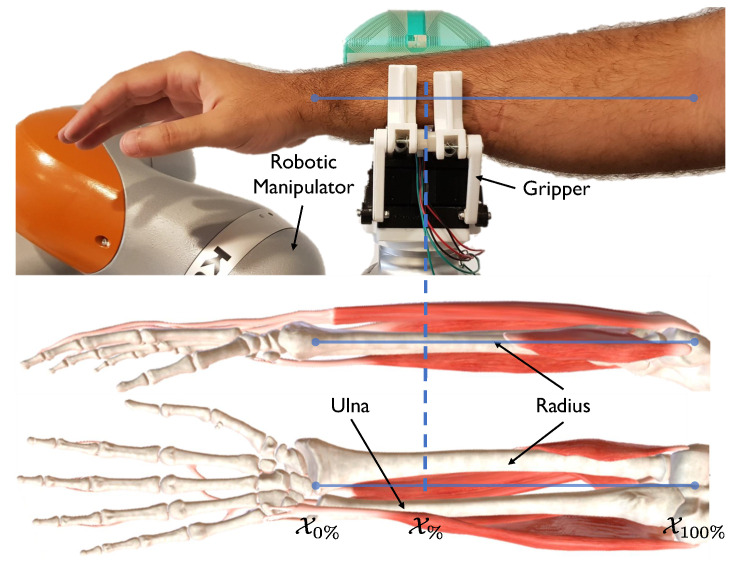
The robotic manipulator with the three-fingered gripper performs a grasp to recognize the location of the grasped forearm. Bones and muscles present different shapes and sizes along the forearm. The 3D images of the musculoskeletal model at the bottom are from BioDigital [32].

This paper presents a novel dataset of a forearm obtained with a gripper that records full haptic perception. The three-fingered gripper contains a high-resolution tactile sensor in a finger and two independent underactuated fingers with proprioceptive sensors to provide kinesthetic data. The gripper performs a squeeze and release process, which approximates human palpation to obtain both tactile and kinesthetic data over time. With this procedure, some characteristics can be obtained, such as size, stiffness, and hard inclusions [22]. Thirteen equally spaced measurements, from the wrist to the elbow, have been recorded, with a total of sixty experiments each (13×60). This dataset provides information about the bones and muscles, whose size and position vary along the forearm, as seen in Figure 1, and could be used as training data in pHRI applications. To illustrate the application of the recorded dataset, we present an estimation of the forearm’s grasping location. A regression approach has been taken into consideration using LSTM neural networks. To the best of our knowledge, this is the first dataset that provides tactile and kinesthetic information about a whole human forearm. This information is relevant in the case of performing a safe upper-limb manipulation to avoid the wrong manipulation that could hurt the human. Moreover, some procedures must be performed in specific parts of the forearm, such as locating sensors to obtain optimal biomedical signal readings or performing medical assistance.

The main contributions of this work are:

- A tactile-kinesthetic dataset obtained with a gripper of a whole human forearm for pHRI applications.
- An example of the use of this dataset with a deep learning fusion-based regression approach, where both tactile and kinesthetic information are utilized to estimate the location of the gripped section on the forearm.

The performance of the proposed neural network is analyzed, providing non-trained examples to the regression approach, and comparing the outputs with the ground truth data. The dataset and code are publicly available in a GitHub repository (https://github.com/fpastorm/Forearm-tactile-kinesthetic-dataset, accessed on 8 November 2022).

This paper is organized as follows: Section 2 presents the experimental setup necessary for the dataset acquisition. Section 3 details the dataset collection process. Section 4 describes how the tactile and the kinesthetic data can be used for a regression approach. The experiments performed are related in Section 5, followed by the results obtained and the discussion of the results in Section 6. Finally, Section 7 includes the conclusions and prospective research work.

## 2. Experimental Setup

The experimental setup used to record the dataset is described in this section. On the one hand, the two-sources haptic gripper is presented, describing the sensors and actuators it contains. On the other hand, the novel-designed device used to obtain the forearm dataset is described.

### 2.1. Underactuated Gripper

**Figure 2:**
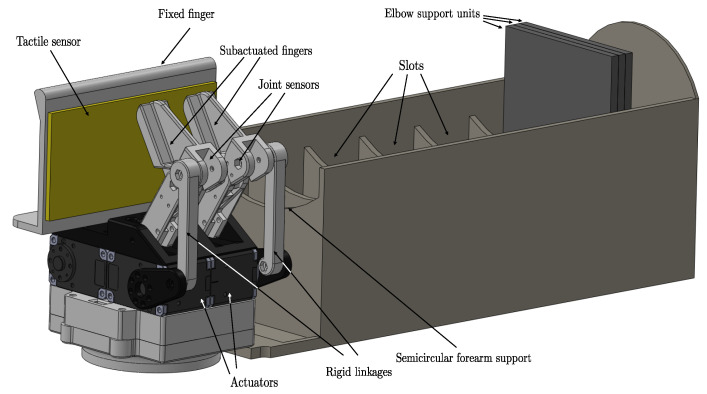
Experimental setup used to obtain a forearm dataset. A description of the elements of the underactuated gripper attached to the forearm measurement device.

The presented robotic hand is a three-fingered gripper used in our previous works [22,27] (see Figure 2). One finger is fixed and contains a tactile sensor over its entire surface. The two reaming fingers are underactuated fingers that are responsible for performing the squeeze-and-release procedure.

The tactile data are collected using the tactile sensor Teskcan (South Boston, MA, USA) 6077 model. The device is a rectangular sensor with 53.3 mm height and 95.3 mm width and with 58×50 tactels or sensels. Moreover, a 3 mm silicon pad covers the entire tactile sensor to enhance the tactile measurements. Table 1 describes the main sensor parameters. A data acquisition system, with the help of the Teskscan real-time SDK, is used to obtain the recorded tactile data in Matlab. The fixed finger with a large tactile sensor is crucial for obtaining the shape of the grasped bodies. Moreover, with this design, the distribution of forces will generally not be applied homogeneously; therefore, in the areas where more significant pressures are applied, some internal characteristics are likely to be obtained.

**Figure 3:**
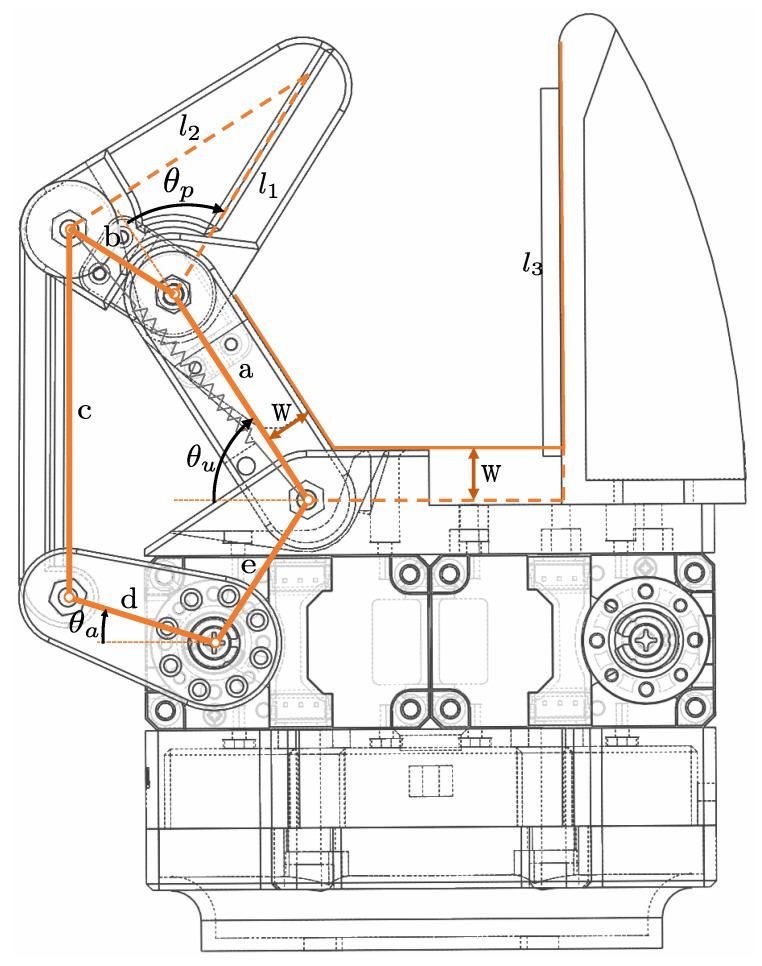
Kinematic design of the gripper, defining the two underactuated fingers parameters and the fixed finger.

Two symmetric independent underactuated fingers complete the entire design of the robotic gripper. Each finger presents two phalanxes to adapt to the shape of the forearm, applying the pressure evenly and on more surfaces than with a conventional single-phalanx finger. The mechanism of an underactuated finger consists of a five-link bar mechanism. As seen in Figure 3, both underactuated fingers have two degrees of freedom ($\theta_{u}$ and $\theta_{p}$), one actuator which is able to apply a given torque ($\tau_{a}$), and a spring that provides rigidness to the whole mechanism when no external forces are applied. Two dynamixel motors (XM430-W210-T) have been used as actuators, and they are controlled with a PWM signal sent to an open-loop torque control mode provided by dynamixel. The passive joint angle between both phalanxes ($\theta_{p}$) is obtained with a potentiometer that acts as an angular sensor (muRata SV01). $\theta_{u}$ is obtained by solving the five-bar mechanism with trigonometric methods [24], knowing $\theta_{a}$, the value of which is provided by the actuator, and $\theta_{p}$. The parameters of the kinematics of the gripper are shown in Table 2. Hereinafter, we will add to the θ angle the subindex *r* (right) or *l* (left), depending on whether we are referring to the right or the left underactuated finger.

### 2.2. Forearm Measurement Device

The forearm measurement device has been designed and built using additive manufacturing technology. Evenly spread measurements are obtained thanks to its design. The device has different elements, which are described below. The first part is the union of the robotic gripper with the device. The second part is the semicircular forearm support, adapted to withstand the whole weight of the forearm. It has also been designed to be comfortable for the human whenever the dataset is being obtained, as its semicircular shape allows the forearm to rest fixed in the device, distributing the weight homogeneously. The device has evenly distributed slots for the placement of the elbow support units. The support units are one centimeter wide.

**Figure 4:**
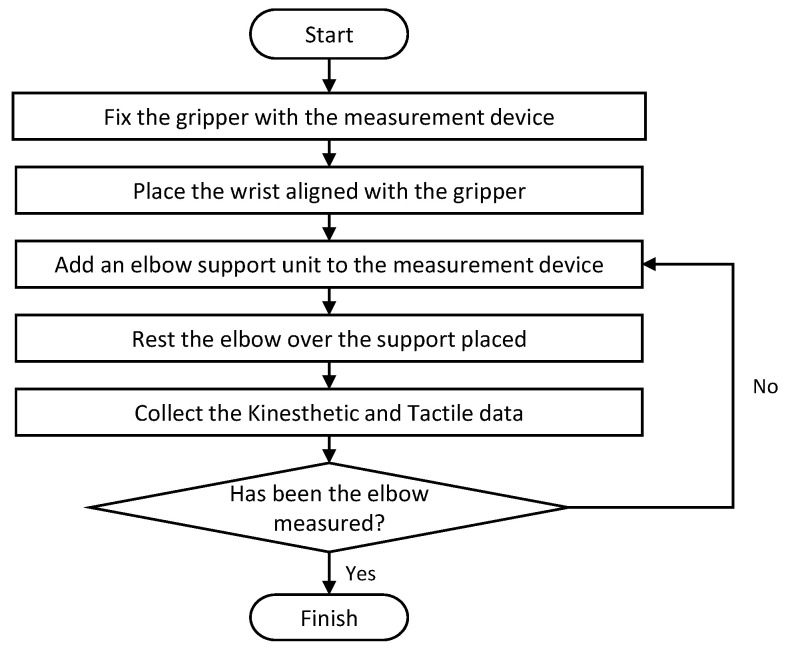
Flowchart of the steps needed to record a full haptic dataset using the forearm measurement device.

The steps that have to be carried out to create a dataset with this device are summarized in the flowchart in Figure 4. First, the gripper is attached to the measurement device, and the forearm is placed on it. The forearm must be aligned with the gripper. Then, an elbow support unit is placed in its corresponding slot so that the back of the elbow is in touch with that element, ensuring that the forearm remains completely stationary. The next step is to perform the data collection process as described in Section 3 for that specific section of the forearm. Then, a new support unit is added to the same slot so that the forearm is moved one centimeter from its previous position once the elbow is in touch with the new support unit. The data collection step is repeated until reaching the elbow.

## 3. Dataset Collection Process

Haptic information can be obtained by multiple approaches, realizing different exploration procedures (EPs) [33]. In the case of in-hand recognition, one of the most common EPs is to measure the shape of a grasped body with the kinesthetic information fingers provide. Another common EP is to perform a palpation procedure to measure the stiffness the body has, and to detect some internal features it might present. Both EPs can be performed at the same time, realizing a squeeze-and-release procedure.

A robotic hand (presented in Section 2) is utilized to perform these two EPs. The squeeze-and-release process is realized by holding the forearm inside the robotic hand and grasping it with the help of the two underactuated fingers. Both motors apply random increased (in the squeeze) and decreased (in the release) torque to simulate human palpation. An initial 10% of the max torque is applied, increasing by 5% every 0.5 s, with a random variation of ±2% until 90% of the max torque is applied. Then, the torque decreases to 10%, similarly as described in the squeeze procedure. The randomness in the torque applied is included considering a human does not always perform palpation in exactly the same way. The kinesthetic information is provided by the two underactuated fingers and the tactile information is obtained by the tactile sensor located in the fixed finger.

We will define $X_{\%}$ as the percentage the forearm grasped, considering the wrist is $X_{0\%}$, and the elbow is $X_{100\%}$, following a linear scale for the intermediate grasps, as seen in Figure 1. Overall, thirteen equally spaced EPs have been performed, from the wrist to the elbow of a right forearm of a subject, obtaining the following dataset ($\mathrm{DS}\in R^{13}$) vector:

(1) $$\mathrm{DS}=\left[X_{1},\cdots ,X_{k},\cdots ,X_{13}\right]=\left[X_{0\%},X_{n\%},X_{2n\%},\cdots ,X_{100\%}\right]$$

**Figure 5:**
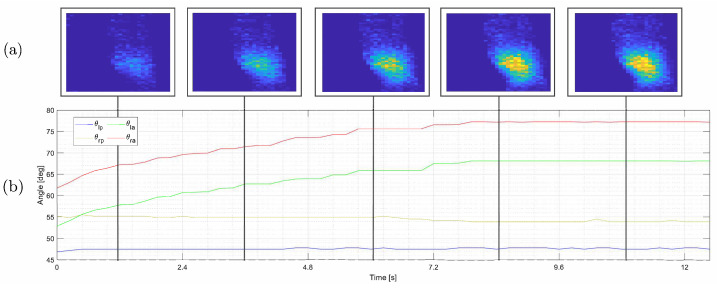
Time sequences of tactile and kinesthetic information are excerpted during the grasping process. A collection of tactile images composing tactile information (**a**). Five tactile images are presented in this figure to demonstrate how the pressure distribution evolves along the sequence, distinguishing the bones and muscles of the forearm. The variation in the joint position of the underactuated fingers’ joints produces kinesthetic information (**b**). Here, $\theta_{la}$ and $\theta_{ra}$ denote the left and right fingers’ actuated joint angles, respectively, while $\theta_{lp}$ and $\theta_{rp}$ denote the left and right fingers’ underactuated joint angles.

Each DS subindex (*k*) is associated with the percentage of the grasped location with the following relationship $\%=n\dot{(}k-1)$, with $n=8.\overset{⏜}{3}$:

A total of 60 iterations have been carried out for each subindex, with a total of 780 experiments. Tactile information is recorded as a time tensor $T\in R^{28\times 50\times K}$, with K=95 being the number of tactile frames obtained in each squeeze-and-release process. An example of some of those tactile frames is represented in Figure 5a. The kinesthetic data recorded are a time matrix $\theta \in R^{4\times I}$, where I=79 represents the number of samples recorded in each squeeze-and-release EP. The time matrix records the position of both actuators ($\theta_{ra},\theta_{la}$) and the angle between the underactuated joints ($\theta_{rp},\theta_{lp}$), as seen in Figure 5b. We will define the roll angle constant, considering it can be estimated and reoriented to a given angle [25]. Therefore, for this dataset, we will define the wrist roll angle as 0 for each measurement.

## 4. Tactile and Kinesthetic Data Fusion for Regression

Physical interaction between robots and human upper limbs is crucial for many applications. For instance, rescue tasks, where a triage must be performed, locating biomedical sensors in specific parts of the forearm where they obtain better locations, or injecting medicine to survivors in critical condition. Other applications, such as assistive robotics, perform robot-initiated upper limb pHrI. Manipulating human upper limbs can be a high-risk task, in the sense we could harm the subject if we lack information about the grasped position and the forearm roll angle. Considering these necessities, in this work, we present a regression method using the haptic dataset to estimate the grasped forearm location ($X_{\%}$). Both tactile and kinesthetic information are trained individually, and then, the outputs are fused to enhance the results, similar to our previous work [27], where we fused the haptic information but for classification purposes.

### 4.1. Neural Networks Structure

**Figure 6:**
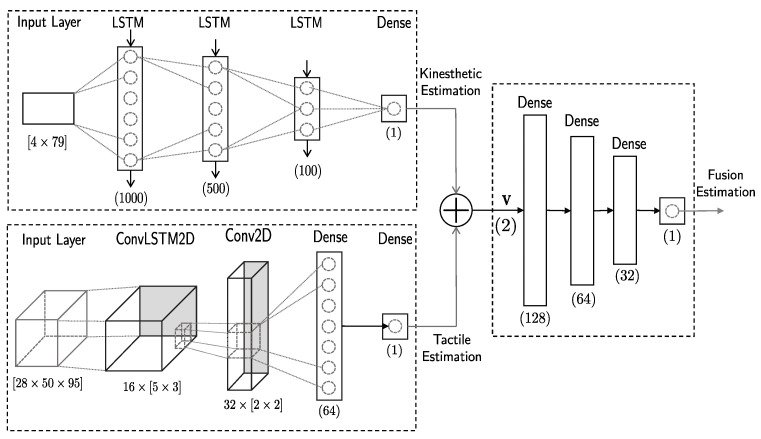
Diagram of the regression neural network structure. Kinesthetic and tactile data are trained separately. The kinesthetic network is defined by three LSTM layers and a fully connected layer. The tactile network is formed by a convolutional LSTM layer with a convolutional layer and two fully connected layers. Both outputs are concatenated and fed to a fully connected layer fusion network that uses both sources of information to improve the output.

A schematic of the three regression neural networks is presented in Figure 6. Both tactile and kinesthetic networks are based on LSTM layers. This type of layer learns long-term dependencies between sequence data obtained in time series and is able to preserve previous information, as demonstrated in various works [27,34]. The squeeze-and-release process provides haptic information with an evident temporal structure; therefore, it seems intuitive to conclude that LSTM networks will perform adequately.

The tactile images time series (T) is used to train the tactile network, which is formed by four layers. It presents a Convolutional LSTM [35] layer (LC=ConvLSTM2D), with a kernel of $16\times \left[5\times 3\right]$ and a hyperbolic tangent (tanh) activation function, followed by a convolutional layer (C=Conv2D) with a kernel of $32\times \left[2\times 2\right]$ and a Rectified Linear Unit (ReLU) activation function. Then, two fully connected layers (F=Dense) with 64 neurons, and ReLU activation function, and a single-neuron and linear activation function, respectively.

The kinesthetic network is fed with the angle time matrix (θ) and is formed by four layers. The first three layers are LSTM layers (L=LSTM), with 1000, 500, and 100 neurons, respectively, to achieve a progressive codification of the input matrix. All of them have tanh as an activation function. The last layer is a single-neuron fully connected layer with a linear activation function.

Considering tactile and kinesthetic estimation outputs may differ, its results are interesting for learning the strengths and weaknesses of each network with a new fusion neural network that uses both sources. Estimation outputs are concatenated, creating a new input matrix $V\in R^{2}$. The fusion network presented is a four-layer fully connected network with 128, 64, 32, and 1 neurons, respectively. All layers have a ReLU activation function, with the exception of the last layer, which has the linear activation function.

### 4.2. Training

To effectively train the tactile and kinesthetic data and then the fusion neural network, let us define two new subsets created from the original dataset (DS):

(2) $$\mathrm{SO}=\left\{{\mathrm{SO}}_{i}\in \mathrm{DS}\;/\;i=2k,k\in Z^{+}\right\}=\left[X_{n\%},X_{3n\%},\cdots ,X_{11n\%}\right]$$

(3) $$\mathrm{SE}=\left\{{\mathrm{SE}}_{l}\in \mathrm{DS}\;/\;l=2k-1,k\in Z^{+}\right\}=\left[X_{0\%},X_{2n\%},X_{4n\%},\cdots ,X_{100\%}\right]$$

$\mathrm{SO}\in R^{6}$ contains the elements of the odd subindex of DS, while $\mathrm{SE}\in R^{7}$ is formed by the even subindex elements of DS. This division of data is not arbitrary and will be discussed in Section 5.

The tactile and kinesthetic networks are trained with the SE subset. On the one hand, the tactile network estimation model is trained with 54 examples for each $X_{\%}$ (90% of the subset), using 20% of the training data for validation. An Adam optimizer function is utilized for training, using the mean squared error as a loss function, with a learning rate of 8 × 10^−4^ over 500 epochs. On the other hand, the kinesthetic network estimation model is trained on the same 54 samples with the same 20% of the training data for validation purposes. An Adam optimizer function is also utilized for training, using the mean squared error as a loss function, with a learning rate of 1 × 10^−5^ over 2000 epochs.

Finally, both tactile and kinesthetic networks are fed with the entire SO subset, and then, as described in Section 4.1, the outputs are used to train the Fusion neural network estimation model. Similarly, as with the tactile and kinesthetic neural networks, 90% of the data has been used for training purposes, with 20% for validation procedures. An Adam optimizer function is also used for training, using the mean squared error as a loss function, with a learning rate of 1 × 10^−6^ over 2500 epochs.

## 5. Experiments

To effectively evaluate the performance of the three neural networks, they will all be fed with non-tested data from the known subset and completely new data from the other subset. Therefore, tactile and kinesthetic neural networks will be tested with the remaining 10% examples from each *l*SE element and with the entirely unknown SO. Similarly, the fusion network will be tested with the remaining 10% of examples from each *i*SO element and with the entirely unknown SE subset. To obtain significant statistical performance metrics, 20 trainings and tests of each regression neural network have been performed. In each of the iterations, random training data and random test data are taken from the 60 experiments, but the tactile data and the kinesthetic data always belong to the same squeeze-and-release process. We will evaluate the performance by analyzing the maximum and minimum estimated values, the amplitude of the 25/75 percentiles, and the median of the estimated values. Moreover, an analysis of the Root Mean Squared Error (RMSE) and the Mean Absolute Error (MAE) of each percentage of the grasped forearm will be performed.

The training and test experiments were performed using the Keras API in an Intel Core i7-8700K computer with 16 GB of RAM, equipped with an NVIDIA RTX 2080Ti graphics processing unit (GPU).

## 6. Results and Discussion

**Figure 7:**
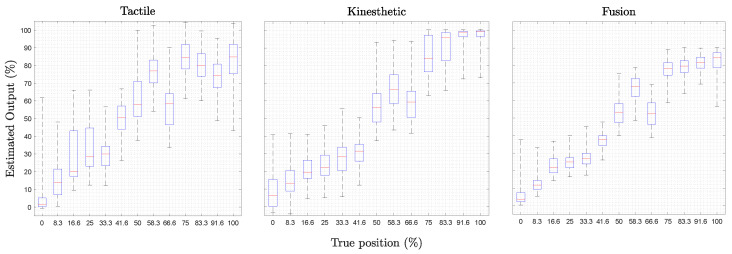
The median, 25/75 percentiles, and the maximum and the minimum estimation output of each $X_{\%}$. The test is presented for the tactile, kinesthetic, and fusion neural networks.

**Figure 8:**
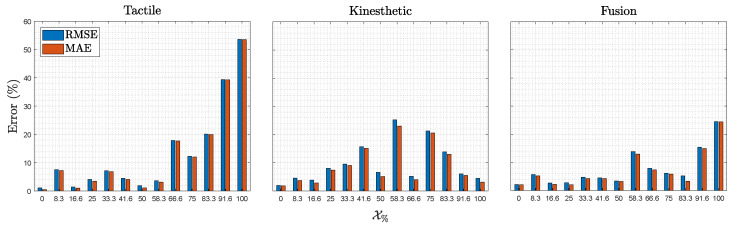
The root mean squared error (RMSE) and the medium absolute error (MAE) achieved for each $X_{\%}$ tested. Both errors are presented for the kinesthetic, the tactile, and the fusion neural networks.

The results of the 20 iterations of experiments are presented in this section. The estimation output values are presented in a box plot for each neural network, as seen in Figure 7. Moreover, the RMSE and the MAE of each percentage of the grasped forearm for each neural network are shown in Figure 8, with Table 3 summarizing the results.

As appreciated in Figure 7, the tactile regression neural network presents the most significant range between the minimum and the maximum output, with an average of 50.87%. On the other hand, the kinesthetic regression neural network presents better results, having an average range of 41.56%. The fusion provides the minor range, with an average of 28.24%. As expected, fusion is the best approach, considering that their results are the most consistent over the 20 iterations. Similarly, the range of the 25/75 percentiles is the highest in the tactile regression neural network, followed by the kinesthetic neural network. Again, the fusion neural network outperforms with the lowest range.

Regarding MAE and RMSE errors, as seen in Table 3, the tactile neural network approach presents the most significant average error. Nevertheless, the results provide a fair estimation of the grasped section and could be utilized in applications that do not require high accuracy. As presented in Figure 8, the accuracy of the estimation in the tactile neural network is very precise from the wrist ($X_{0\%}$) until approximately half of the forearm ($X_{58.3\%}$). Then, the accuracy decreases abruptly until the elbow ($X_{100\%}$). This occurs due to the fact that in the zone between the wrist and the middle of the forearm, the bones are closer to the skin; therefore, when performing the squeeze-and-release process, the tactile sensor obtains a large amount of internal information. However, in the area from the middle of the forearm to the end of the elbow, the bone is deeper with respect to the skin surface; thus, the sensor finds less information that differentiates the forearm sections. The kinesthetic neural network outperforms the average results of the tactile neural network; thus, it is also possible to realize precise estimations. The kinesthetic estimation presents its best results in the zones close to the wrist and the elbow. However, it lacks the capacity to recognize half of the forearm zone. Kinesthetic data obtains information about the shape of the forearm; therefore, we can assume that the shape of both the wrist and elbow are sufficiently different. However, it is intuitive to conclude that the middle part of the forearm presents similar shapes, considering the estimation is worse in that zone. Lastly, the fusion estimator presents the best average error. The error is low in almost every $X_{\%}$, except in $X_{58.3\%}$ and $X_{100\%}$, coinciding with the highest errors of the kinesthetic and tactile neural networks, respectively. The robustness of the fusion network is remarkable. Although some error in the estimation is shown, no disparate outputs are presented.

Even though the results are satisfactory, they could be improved, including a more extensive dataset incrementing the thirteen grasps measurements and increasing the sixty experiments realized on each measurement. However, gathering a significant amount of tactile and kinesthetic data is a big investment. Various techniques for this challenge could be used, such as sim-to-real approaches that pre-train the models using simulated data; or employing generative adversarial networks (GANs) or variational autoencoders to produce new data that are equivalent to that received by the real sensor. It is also important to remark that this is training performed with a dataset formed from only one subject’s information; thus, a satisfactory performance in other subjects is not ensured.

## 7. Conclusions

In this work, a haptic database of a human upper limb was presented. Thirteen equally spaced measures have been obtained from the wrist to the elbow of the right forearm. The data have been recorded by a three-fingered robotic gripper, with a fixed finger containing a tactile sensor to perceive tactile information and two underactuated fingers that perform the grasping procedure and record proprioceptive information.

Moreover, an application using the collected dataset has been designed. An estimation of the grasped forearm position was presented, fusing the haptic data in a neural network approach. Tactile and kinesthetic information have been trained separately, and their outputs were fed to a new fusion neural network that has enhanced the results.

Future research shall consider the addition of tactile sensors to the underactuated fingers to improve the sensing capabilities of the whole gripper, including different forearm datasets, considering male and female subjects and different forearm profiles, taking into account various factors, such as muscle, fat percentage, or wingspan. Some data augmentation procedures could also be applied to increase the provided dataset, using flipping and scale techniques. Retraining this new dataset could lead to the regression fusion neural network being able to estimate grasped sections of left hands grasped symmetrically and sections of grasped forearms of different subjects. However, these assumptions must be handled with caution and studied in depth.

## Figures and Tables

**Table 1:** Features of Tekscan 6077 tactile sensor and silicone pad.

Parameter	Description
Number of tactels	1700
Max. pressure	34 KPa
Temperature range	$-40{\;}^{\circ }$C to $+60{\;}^{\circ }$C
Thickness	0.102 mm
Tactels density	27.6 tactels/cm${}^{2}$
Silicone pad	Ecoflex${}^{\mathrm{TM}}$ 00-30

**Table 2:** Parameter values for the kinematic model of the gripper with underactuated fingers.

Parameter	Value	Parameter	Value
a	40 mm	$l_{1}$	40 mm
b	20 mm	$l_{2}$	44.72 mm
c	60 mm	$l_{3}$	70 mm
d	25 mm	*w*	10 mm

**Table 3:** RMSE and MAE% error of the entire test for each neural network.

	Tactile	Kinesthetic	Fusion
RMSE	13.42	9.74	7.61
MAE	13.09	8.81	7.08

## Abbreviations

The following abbreviations are used in this manuscript:

HRI	Human–Robot Interaction
SAR	Search And Rescue
pHRI	physical Human–Robot Interaction
GA	Genetic Algorithms
LSTM	Long Short-Term Memory
EPs	Exploration Procedures
ConvLSTM2D	Convolutional LSTM
Conv2D	Convolutional
tanh	hyperbolic tangent
ReLU	Rectified Linear Unit
RMSE	Root Mean Squared Error
MAE	Mean Absolute Error
GPU	Graphics Processing Unit
GANs	Generative Adversarial Networks
